# MERS-CoV Spike Protein Vaccine and Inactivated Influenza Vaccine Formulated with Single Strand RNA Adjuvant Induce T-Cell Activation through Intranasal Immunization in Mice

**DOI:** 10.3390/pharmaceutics12050441

**Published:** 2020-05-10

**Authors:** Hye-Jung Kim, Hye Won Kwak, Kyung Won Kang, Yoo-Jin Bang, Yu-Sun Lee, Hyeong-Jun Park, Jae-Yong Kim, Hyo-Jung Park, Kyung-Ah Hwang, Sang-Myeong Lee, Jae-Hwan Nam

**Affiliations:** 1 Department of Biotechnology, The Catholic University of Korea, Bucheon 14662, Korea; kkim0228@naver.com (H.-J.K.); 4111aqsw@naver.com (H.W.K.); byj106@naver.com (Y.-J.B.); leeyusun9393@naver.com (Y.-S.L.); kocom123@naver.com (H.-J.P.); wodyd202@gmail.com (J.-Y.K.); winter80@empal.com (H.-J.P.); 2 Division of Biotechnology, The Jeonbuk National University, Iksan 54596, Korea; gp1900@naver.com (K.W.K.); leesangm@jbnu.ac.kr (S.-M.L.); 3 Department of Research and Development, SML Genetree Research, Seoul 06741, Korea; kahwang@genetree.co.kr

**Keywords:** intramuscular immunization, intranasal immunization, RNA adjuvant, protein-based vaccine, inactivated vaccine, Middle East respiratory syndrome coronavirus (MERS-CoA), influenza virus

## Abstract

The effectiveness of vaccines is enhanced by adding adjuvants. Furthermore, the selection of an inoculation route depends on the type of adjuvant used and is important for achieving optimum vaccine efficacy. We investigated the immunological differences between two types of vaccines—spike protein from the Middle East respiratory syndrome virus and inactivated influenza virus vaccine, in combination with a single-stranded RNA adjuvant—administered through various routes (intramuscular, intradermal, and intranasal) to BALB/c mice. Intramuscular immunization with the RNA adjuvant-formulated spike protein elicited the highest humoral immune response, characterized by IgG1 and neutralizing antibody production. Although intranasal immunization did not elicit a humoral response, it showed extensive T-cell activation through large-scale induction of interferon-γ- and interleukin-2-secreting cells, as well as CD4+ T-cell activation in mouse splenocytes. Moreover, only intranasal immunization induced IgA production. When immunized with the inactivated influenza vaccine, administration of the RNA adjuvant via all routes led to protection after viral challenge, regardless of the presence of a vaccine-specific antibody. Therefore, the inoculation route should depend on the type of immune response needed; i.e., the intramuscular route is suitable for eliciting a humoral immune response, whereas the intranasal route is useful for T-cell activation and IgA induction.

## 1. Introduction

Vaccines are currently one of the most effective and practical ways to protect the human body against various infectious diseases [1,2]. Vaccines induce an antigen-specific immune response, which elicits a stronger secondary immune response after the individual is exposed to the pathogen again. Therefore, vaccinations prevent infectious disease because the pathogen-specific immune system, which has been previously trained, can eradicate pathogenic antigens when exposed to live pathogens [3,4]. Diverse routes have been used for vaccine inoculation, to maximize their effectiveness, and are determined by considering the characteristics of the vaccine, physiological environment of the site of injection, and purpose of vaccination, such as for prophylactics or therapeutics use [5,6]. Currently, only the Bacillus Calmette–Guérin vaccine is injected intradermally. Vaccines are administered subcutaneously for measles and yellow fever and are typically administered intramuscularly for hepatitis B, polio, tetanus, and diphtheria. Additionally, the FDA-approved influenza vaccine, FluMist Quadrivalent, is administered intranasally [7,8].

Preventive vaccines currently used in clinical practice are mainly protein-based. Protein vaccines induce humoral immune responses but weakly induce cellular immune responses, particularly T-cell induction. Thus, an effective strategy is to induce stronger immunity by using multiple immunizations or adjuvants to enhance the immune response [9]. Some adjuvants have been approved by the FDA, such as alum, MF59, AS03, CpG 1018, and others [10,11,12,13]. Aluminum potassium sulfate, referred to as alum, induces a strong innate immune response through the influx of neutrophils, eosinophils, natural killer cells, CD11b+ monocytes, and dendritic cells at the injection site [11]. However, alum induces a weak T-cell-mediated immune response and weak maturation of antigen-presenting cells (APC). Thus, various adjuvants have been developed to overcome this issue [14].

Exogenous mRNA is intrinsically immunostimulatory because it is recognized by innate immune receptors on various cell surfaces, endosomes, and the cytoplasm [15]. Among them, single-stranded RNA (ssRNA) can act as an agonist of Toll-like receptor 7/8 (TLR-7/8) and retinoic acid-inducible gene I to induce an innate immune response [16]. Therefore, ssRNA can be used as an adjuvant to increase the efficiency of recombinant protein-based vaccines. In mouse experiments, RNA adjuvant was shown to balance Th1/Th2 immune responses through intramuscular administration [17]. Additionally, in mice immunization experiments, ssRNA was safe as an adjuvant, causing no tissue damage, from a histopathological perspective, and causing no weight loss or abnormal behavior [18].

We previously developed a new ssRNA platform and studied the effects of adjuvants [17,19]. The ssRNA adjuvant was derived from the cricket paralysis virus intergenic region internal ribosome entry site [19]. In this study, we formulated an RNA adjuvant with spike (S) protein-subunit vaccine for Middle East respiratory syndrome (MERS) coronavirus (CoV) and inactivated the influenza virus vaccine to investigate how the immune response differs according to the route of inoculation. Finally, we assessed which administration routes are most effective for the RNA-adjuvant-formulated vaccination.

## 2. Materials and Methods

### 2.1. Mice

Six-week-old female BALB/c mice were purchased from Dae-Han Bio-Link (Chungbuk, Korea). Mice were housed at the Catholic University of Korea, under specific-pathogen-free conditions with a standard light cycle (12 h light/dark cycle) and handled according to protocols approved by the Catholic University of Korea. The animal facility at the Catholic University of Korea is fully accredited by the Korean Association for Laboratory Animals (2018-027, 24 August 2018). All mice experimental procedures conducted in this study followed the guidelines of the Institutional Animal Care and Use Committee of the Catholic University of Korea (CUK-IACUC-2018-027).

### 2.2. Vaccines

Soluble MERS S protein vaccine was provided by the International Vaccine Institute (Seoul, Korea). The MERS S protein expressed in insect cells has no transmembrane domain and comprises the first 1296 amino acids of the MERS-CoV EMC/2012 strain (Genbank #AFS88936.1). The inactivated influenza virus vaccine is a cell-based vaccine (SkyCell Flu, from SK Bioscience, Seoul, Korea). The SKYCellflu^®^ for the 2018–2019 Northern Hemisphere season, which is a quadrivalent influenza vaccine consisting of A/Michigan/45/2015(NYMC X-275) for A/H1N1, A/Singapore/INFIMH-16-0019/2016 (IVR-186) for A/H3N2, B/Phuket/3073/2013 for B/Yamagata, and B/Maryland/15/2016 for B/Victoria, was used as the seasonal IIV.

### 2.3. In Vitro Transcription and RNA Purification

The DNA platform was designed by using the intergenic region internal ribosome entry site and SV40 late-polyadenylation signal sequences [19]. DNA templates were linearized with Not Ⅰ (Enzynomics, Daegeon, Korea). In Vitro transcription was performed, using the EZ T7 High Yield In Vitro Transcription Kit (Enzynomics), as reported in [19]. 

### 2.4. Immunization

For MERS S protein vaccine studies, the mice were immunized twice, at two-week intervals, with the following formulations: 1 µg MERS S protein vaccine with/without 20 µg RNA adjuvant, and 24 µg alum (Brentanne, Frederikssund, Denmark). For influenza vaccine studies, the mice were immunized twice per week, at two-week intervals, with 0.6 µg inactivated influenza vaccine, with/without 20 µg RNA adjuvant. Mice were injected intranasally, intramuscularly, or intradermally in the upper-thigh position. For intranasal injection, the volume was limited to 0.02 mL or less. The mouse was held in the supine position, with the head elevated [20].

### 2.5. Challenge with Influenza Virus

Each mouse was infected with 1.0 × 10^3^ plaque-forming units (PFU) of influenza virus (A/H1N1/California/04/09), intranasally. Influenza viruses were provided by Professor Baik-Lin Seong (Yonsei University, Seoul, South Korea).

### 2.6. Serum Collection

Serum was collected from MERS S protein-immunized mice at two and four weeks and from influenza vaccine-immunized mice at two and three weeks after the first vaccination. Serum samples were collected from the facial vein, using an 18-G needle. The samples were stored at −80 °C, until use.

### 2.7. Bronchoalveolar Lavage Fluid (BALF) Collection

Bronchoalveolar lavage (BAL) was performed on sacrificed mice by flushing the airway compartment with 1 mL PBS via inserting a 22-G catheter into the trachea of mice. The fluids obtained from BAL (BALF) were centrifuged at 800 × *g* for 10 min at 4 °C.

### 2.8. Enzyme-Linked Immunosorbent Assay (ELISA)

Antigen-specific IgG1 and IgG2a in mouse serum and antigen-specific IgA in the mouse BALF were measured by ELISA. The 96-well plates (Corning, Inc., Corning, NY, USA) were coated with 50 ng/well of MERS S protein and 100 ng/well of influenza vaccine and incubated overnight at 4 °C. After incubation, the wells were blocked with 200 µL blocking buffer (PBS-1% bovine serum albumin) for 1 h at room temperature. Diluted serum samples (1:100 dilution for measurement of IgG1 and IgG2a) and BALF solutions were added to the plates and incubated for 1 h at room temperature. After incubation, the wells were washed three times with 200 µL PBS-T (PBS-0.05%, Tween 20). The anti-mouse IgG1, IgG2a, and IgA-horseradish peroxidase (Invitrogen, Carlsbad, CA, USA; Novus Biologicals, Littleton, CO, USA; and Bethyl Laboratories, Montgomery TX, USA, respectively)-conjugated antibodies, diluted 1/5000 in PBS, were added to the plate and incubated for 1 h at room temperature. After three washes with PBS-T, 3,3′,5,5′-tetramethylbenzidine substrate (Invitrogen) was added and incubated for 15 min and then 2N H_2_SO_4_ was used to stop the reaction. The O.D. values were measured at 450 nm, using a GloMax Explorer Multimode Microplate Reader (Promega, Madison, WI, USA). To measure cytokines in the splenocyte culture supernatants, mouse splenocytes were collected and isolated from an immunized mouse. Splenocytes were seeded at a density of 5 × 105 cells per well (96-well plate). To re-stimulate the splenocytes, 500 ng/well of MERS S protein was added to the culture medium for two days, after which the medium was assessed with ELISA. The concentrations of interferon γ (IFN-γ), interleukin-2 (IL-2), IL-6, and tumor necrosis factor α (TNF-α) were detected with ELISA kits (Invitrogen; Thermo Fisher Scientific Inc., Waltham, MA, USA), according to the manufacturer’s instructions. The concentrations of these cytokines were calculated according to standard curves, and the obtained results are shown as the amount (pg) of IFN-γ, IL-2, IL-6, and TNF-α per mL of supernatant.

### 2.9. Plaque-Reduction Neutralization Test for Middle East Respiratory Syndrome Coronavirus (MERS-CoV)

The serum samples from vaccinated mice were inactivated at 56 °C for 30 min. The samples were serially diluted from 1/40 to 1/640 with serum-free medium. The virus–serum mixture was prepared by mixing 125 PFU MERS-CoV with the diluted serum samples and incubated at 37 °C for 1 h. The virus–antibody mixture was inoculated into Vero cells. The plates were incubated for 1 h at 37 °C in 5% CO_2_. After virus adsorption, agar overlay medium was added, and the plates were incubated at 37 °C in 5% CO_2_ for four days. The cells were stained with 0.4% crystal violet solution (Sigma, St. Louis, MO, USA). Plaques were counted with the naked eye. The percentage neutralization represented the reduction value, which was calculated as 100× the number of plaques in the 100 PFU virus-infected well/ number of plaques in the virus–serum mixture-infected well.

### 2.10. Enzyme-Linked Immunospot (ELISPOT)

Splenocytes from immunized mice were stimulated with 500 ng/well of antigens for 48 h at 37 °C. ELISPOT was performed to detect IL-2- and IFN-γ-secreting T-cells according to manufacturer’s instructions (Mabtech, Stockholm, Sweden).

### 2.11. Flow Cytometry

For splenocyte surface staining, the following antibodies were incubated for 15 min at room temperature: CD11b (Clone M1/70, BioLegend, San Diego, CA, USA), CD45R/B220 (Clone RA3-6B2, BD Biosciences, Franklin Lakes, NJ, USA), F4/80 (Clone BM8, eBioscience, San Diego, CA, USA), CD86 (Clone GL1, BD Biosciences), and CD11c (Clone N48, eBioscience). The stained splenocytes were fixed with 1% paraformaldehyde. After staining, a FACSCanto II flow cytometer (BD Biosciences) and FlowJo (Tree Star, Inc., Ashland, OR, USA) were used for analysis. For polyfunctional T-cells, isolated splenocytes were re-stimulated with 500 ng/well MERS S protein. Brefeldin A (GolgiPlug, BD Biosciences) and monensin (GolgiStop, BD Biosciences) were incubated for 2 h. After another 10 h incubation, the splenocytes were stained with ethidium monoazide (Sigma) and then stained with anti-CD19-BV510, anti-CD3-APCeF710, anti-CD4-PerCPeF710, and anti-CD8-PEcy7 (Clones 1D3, 145-2C11, GK1.5, and 53-6.7, eBiosciences). The stained cells were permeabilized using Cytofix/Cytoperm kit (BD Biosciences) and then stained with anti-IFN-γ-APC, anti-TNF-a-FITC, and anti-IL-2-PE (Clones XMG1.2, MP6-XT22, JES6-5H4, eBiosciences). The stained cells were fixed with 1% paraformaldehyde, analyzed using an LSRII flow cytometer (BD Biosciences), and T-cells positive for the various combinations of cytokines and degranulation were analyzed and quantified, using a Boolean gating function in FlowJo (Tree Star).

### 2.12. Real-Time PCR for Virus Titration

Total RNA was isolated from the lungs, using TRIzol reagent (Favorgen, Ping-Tung, Taiwan), and from BALF, using QIAamp MinElute virus spin kit (Qiagen, Hilden, Germany), and cDNA was synthesized by using a ReverTra Ace QPCR RT Master Mix (Toyobo, Osaka, Japan). The total reaction volume of 25 μL, containing either 10 μL of template RNA, standard control, or negative control and 12.5 μL of 2× SuperScript III platinum master mix, 0.5 μL SuperScript III Taq polymerase, 2 μL of forward primer (10 μM), 2 μL reverse primer (10 μM), and Dual-labeled Probe (5 pmol) mix was added to the real-time PCR plate. Real-time PCR was conducted in Bio-Rad thermocycler. The PCR conditions were 30 min at 50 °C and 5 min at 95 °C, followed by 45 cycles of 20 s at 95 °C and 1 min at 55 °C. For virus detection, we used two pairs of influenza virus-specific primers and TaqMan probes. These primer sets and probes were designed from conserved matrix gene region of influenza A virus (influenza A virus universal forward primer, 5′-GACCRATCCTGTCACCTCTGAC-3′; reverse primer, 5′-AGGGACTTYTGGACAAAKCGTCTA-3′; probe, 5′-FAM-TGCAGTCCTCGCTCACTGGGCACG-BHQ1).

### 2.13. Statistical Analysis

To assess significant differences between two groups, a Student’s *t*-test was used. Differences were considered significant at *p* < 0.05.

## 3. Results

### 3.1. Intramuscular Inoculation with RNA Adjuvant Formulated Protein Vaccine is Effective to Induce the Humoral Immune Response

**Figure 1:**
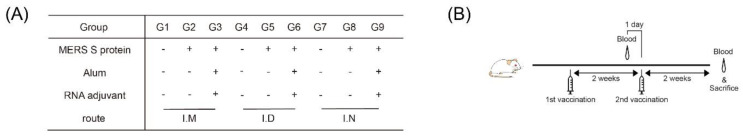
Group design and immunization schedule for Middle East respiratory syndrome (MERS) spike (S) protein. BALB/c mice were intramuscularly (I.M.), intradermally (I.D.), and intranasally (I.N.) immunized, at two-week intervals, with two doses of MERS S soluble protein vaccine with/without RNA adjuvant or alum. (**A**) Overall study design. (**B**) Schedule for mice vaccination.

**Figure 2:**
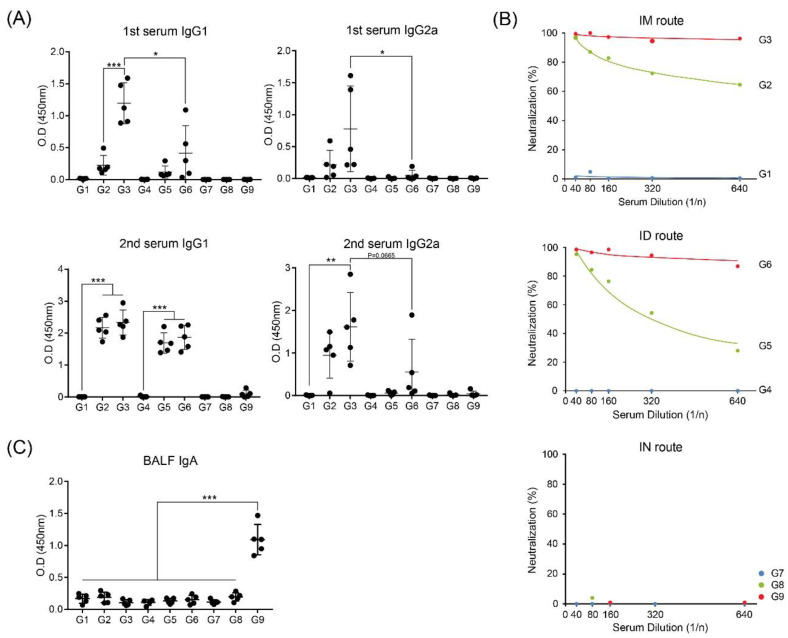
Immunization of RNA adjuvant with MERS S protein induces a humoral immune response. (**A**) MERS S protein-specific IgG1 and IgG2a levels were measured by ELISA. Serum was collected two and four weeks after first immunization. (**B**) MERS-CoV-specific neutralizing antibody (NAb) was determined by plaque-reduction neutralizing assay. Serum was collected two weeks after the second immunization, and each serum was pooled (*n* = 5) and serially diluted from 1/40 to 1/640. (**C**) MERS S protein-specific IgA levels were measured by ELISA in bronchoalveolar lavage fluid (BALF). BALF was collected two weeks after the second immunization. *, *p* < 0.05; **, *p* < 0.005; ***, *p* < 0.001.

To investigate the differences between the inoculation routes with the RNA-adjuvant-formulated protein vaccine candidate, we immunized wild-type BALB/c mice, with or without mixing alum and RNA adjuvant in MERS S protein. (Figure 1A). Immunization was performed twice, at two-week intervals, and autopsy was performed at two weeks after the last immunization. (Figure 1B). At two weeks after the first immunization, IgG1 (indicated Th2 response) in G3, which was immunized intramuscularly with MERS S protein, alum, and RNA adjuvant, showed the highest level. Intradermal inoculation of G6 showed lower IgG1 level than G3 but relatively higher than G2 and G5 immunized only with MERS S protein. At two weeks after the second immunization, G2, G3, G5, and G6 showed similar IgG1 levels, regardless of the immunization routes and RNA adjuvant. In contrast, regardless of boosting, all intranasal injection groups, G7–G9, did not induce any IgG1 levels (Figure 2A). IgG2a (indicated Th1 response) showed the highest levels in the intramuscularly injected group G3 after both the first and second immunizations, whereas IgG2a of G6, which was intradermally immunized with MERS S protein, alum, and RNA adjuvant, like G3, was slightly elevated after boosting immunization. IgG2a levels in all groups inoculated through the intranasal route were not induced by as much as IgG1 levels (Figure 2A). 

Neutralizing antibody (NAb) levels were similarly high in G3 (intramuscular route) and G6 (intradermal route) at two weeks after the second immunization (Figure 2B). However, G2 (intramuscularly immunized with only MERS S protein) showed higher NAb levels than G5 (intradermally immunized with only MERS S protein) (Figure 2B). G2 and G5, which were immunized only with MERS S protein, produced lower NAb levels than those of G3 and G6, which were immunized with MERS S protein, alum, and RNA adjuvant, in agreement with a previous report [17]. As expected, no intranasally immunized groups produced NAbs (Figure 2B).

Interestingly, IgA levels in BALF showed induction only in G9, which was intranasally immunized with MERS S protein, alum, and RNA adjuvant, at two weeks after the second immunization. No other groups (G1–G8) induced increases in IgA levels (Figure 2C).

### 3.2. Intramuscular Inoculation is the Most Effective to Induce NAb, Regardless of Vaccine Components

To investigate whether the effectiveness in intramuscular inoculation in induction of humoral immune response depended on the presence of RNA adjuvant, we immunized mice with various vaccine components through the intramuscular and intradermal routes, using the same immunization schedule as shown in Figure 1B (Supplementary Figure S1A,B). As expected, intramuscular immunization induced higher NAb titers than intradermal immunization compared to identical vaccine components, such as G5 vs. G10; G4 vs. G9; G3 vs. G8; and G2 vs. G7 (Supplementary Figure S1C). Interestingly, G5, which was intramuscularly immunized with MERS S protein, alum, and RNA adjuvant, showed the highest NAb titers. Thus, effective NAb induction was dependent on the inoculation route, regardless of the vaccine components.

### 3.3. Intranasal Immunization of MERS S Protein Enhances CD4+ T-Cell Function 

**Figure 3:**
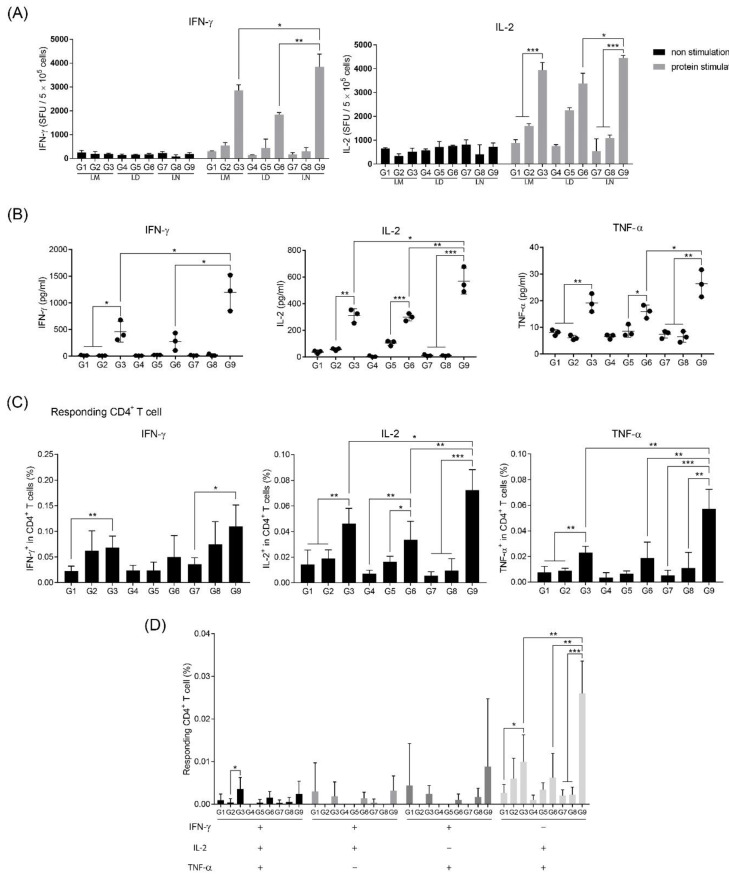
Analysis of T-cell activation after immunization with the RNA adjuvant and MERS S protein vaccine. (**A**) The population of MERS S protein-specific cells secreting interferon-γ (IFN-γ) and interleukin-2 (IL-2) were quantified by using ELISPOT assay, after treatment with MERS S protein in cultured splenocytes from immunized mice. (**B**) Cytokine levels in splenocyte supernatants stimulated with MERS S protein from immunized mice were measured, using ELISA. (**C**) IFN-γ, IL-2, and tumor necrosis factor α (TNF-α)-producing CD4+ T-cells in splenocytes were counted by flow cytometry. (**D**) IFN-γ, IL-2, and TNF-α producing polyfunctional CD4+ T-cells in splenocytes were analyzed by flow cytometry. *, *p* < 0.05; **, *p* < 0.005; ***, *p* < 0.001.

To investigate the difference in activation of T-cells according to the vaccine-administration route, the frequency of IFN-γ- and IL-2-secreting cells from immunized mouse splenocytes was identified after stimulating with MERS S protein, because IFN-γ and IL-2 are secreted from activated and differentiated T-cells [21]. All groups had the same vaccine components and immunization schedule as shown in Figure 1A,B. At two weeks after the second immunization, splenocytes obtained by autopsy were re-stimulated with MERS S protein. Regardless of the immunization route, all groups immunized with the MERS S protein, alum, and RNA adjuvant (G3, G6, and G9) contained more IFN-γ- and IL-2-secreting cells than the other groups (Figure 3A). Interestingly, although G9 did not produce any NAbs (Figure 2B), it showed the highest levels of IFN-γ- and IL-2-secreting cells (Figure 3A). These trends were confirmed by cytokine ELISA of the supernatants of splenocytes after stimulation with MERS S protein. As expected, G9 induced the highest levels of IFN-γ, IL-2, and TNF-α, which are known to act as co-stimulators of T- and B cells (Figure 3B).

To analyze T-cell activation, we performed flow cytometry to identify CD4+ and CD8+ T-cell activation. BALB/c mice were immunized, as shown in Figure 1, and cultured splenocytes were re-stimulated with MERS S protein. IFN-γ-, IL-2-, and TNF-α-secreting CD4+ T-cells of G3, G6, and G9 (different routes, but with the same contents consisting of RNA adjuvant, alum, and MERS S protein as protein-based vaccine candidate) significantly increased compared to other groups without RNA adjuvant (Figure 3C). Specifically, intranasal immunization showed the highest induction (Figure 3C). Moreover, we observed elevations in the number of polyfunctional CD4+ T-cells in G9 (Figure 3D). In contrast to these trends in CD4+ T-cells, there was nearly no significant difference between the groups in CD8+ T-cells (data not shown). These results suggest that the immunization of RNA adjuvant with alum increases CD4+ T-cell induction.

### 3.4. RNA Adjuvant Induces Antigen-Presenting Cells, Especially Dendritic Cells

**Figure 4:**
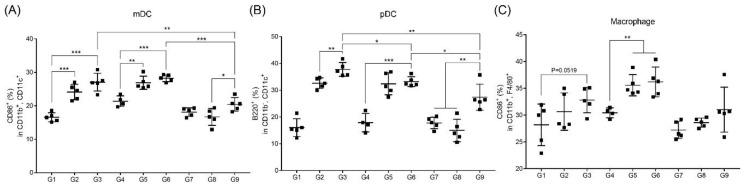
RNA adjuvant activates APCs, particularly dendritic cells (DC). (**A**) Activated myeloid DCs (mDC) (CD11b+CD11c+CD86+), (**B**) plasmacytoid DCs (pDC) (CD11b-CD11c+B220+), and (**C**) macrophages (CD11b+F4/80+CD86+) in splenocytes were counted by flow cytometry. *, *p* < 0.05; **, *p* < 0.005; ***, *p* < 0.001.

As pathogens invade, APCs—particularly professional APCs such as macrophages and dendritic cells (DCs)—take up pathogens and then present the pathogen epitope to immune cells, such as T-cells in spleen or lymph nodes via major histocompatibility complex molecules, causing an immune response [22,23]. We investigated the activation of DCs and macrophages from splenocytes after immunization in BALB/c mice, as shown in Figure 1, by flow cytometry. Myeloid and plasmacytoid DCs (mDC and pDC) of G3, G6, and G9, which were immunized with MERS S protein, alum, and RNA adjuvant showed higher induction compared to the groups without alum (G1, G4, and G7) (Figure 4A,B). G2 and G5, which were immunized with only S protein through the intramuscular and intradermal routes, also showed the induction of mDCs and pDCs similar to G3 and G6. However, in the groups that received intranasal immunization, only G9 (S protein, alum, and RNA adjuvant) showed induction of mDCs and pDCs compared to that of G8 (Figure 4A,B). Macrophages were clearly activated when injected intradermally (G5 and G6 vs. G4). The remaining groups (G3 and G9) appeared to activate the macrophages more than G1 and G7 (Figure 4C). Overall, the activation trends in macrophages were similar to those of DC but were not significant.

### 3.5. RNA Adjuvant Protects Against Viruses

**Figure 5:**
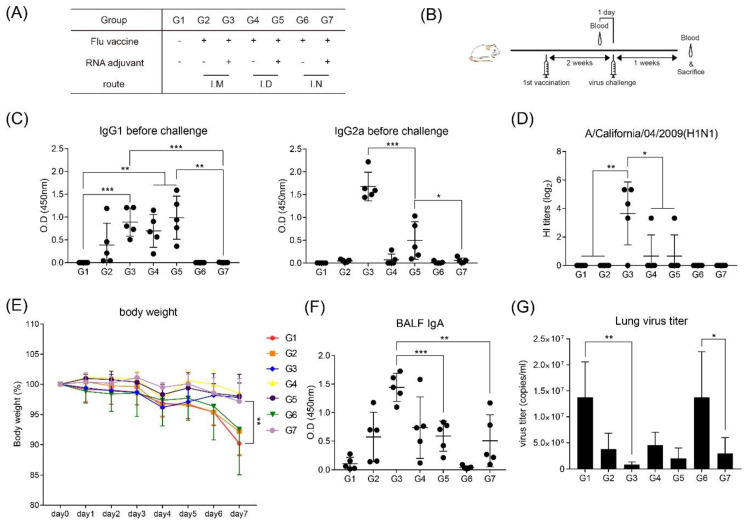
RNA adjuvant formulated influenza vaccine induces humoral immune response and provides protection against viral infections. BALB/c mice were intramuscularly, intradermally, and intranasally immunized with inactivated influenza vaccine with/without RNA adjuvant. (**A**) Overall study design. (**B**) Immunization and virus challenge schedule. (**C**) Influenza vaccine-specific IgG1 and IgG2a levels were measured by ELISA. Serum was collected two weeks after first immunization. (**D**) Hemagglutination inhibition (HI) titer measured by HI assay method. Mice serum was obtained two weeks after first immunization and serially diluted from 1/10 to 1/1280. (**E**) Measurement of weights changes after virus challenge. (**F**) Influenza-vaccine-specific IgA levels were measured by ELISA. BALF solution was collected one week after influenza virus challenge. (**G**) Virus titer for influenza virus was measured by real-time PCR. Lung tissue was obtained one week after influenza virus challenge. *, *p* < 0.05; **, *p* < 0.005; ***, *p* < 0.001.

**Figure 6:**
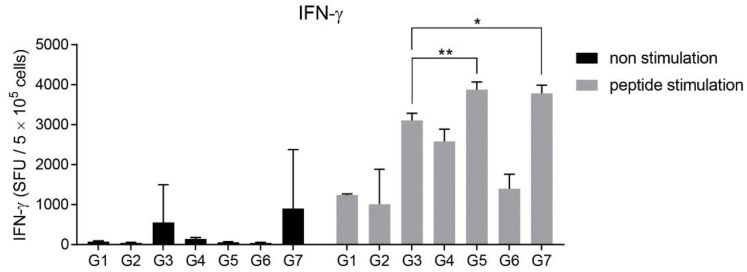
Induction of influenza-specific IFN-γ releasing cells. The population of influenza-vaccine-specific cells secreting IFN-γ was quantified by ELISPOT assay. *, *p* < 0.05; **, *p* < 0.005.

Immunization with or without inactivated influenza vaccine and RNA adjuvant through various routes was performed, to measure pathogen defense immunity. BALB/c mice were challenged with the virus two weeks after the first immunization (Figure 5A,B). When measured for antibody titers, the levels of IgG1 and IgG2a appeared to be similar to those after immunization with the MERS S protein. For IgG1, intramuscular and intradermal injections showed high values. In contrast, the intranasal injection groups were free of antibodies with (G7) or without (G6) RNA adjuvant. IgG2a showed high levels in the group immunized with the RNA adjuvant (G3 and G5), with the highest induction observed following intramuscular injection (Figure 5C). The same trend was observed when the HI titer was measured. Intramuscular immunization with influenza vaccine and RNA adjuvant (G3) resulted in the highest HI titer (Figure 5D). Groups immunized with RNA adjuvant and vaccine (G3, G5, and G7) showed a lower weight change than in the negative control group G1 (Figure 5E). However, IgA measured in BALF after virus challenge was highest following intramuscular immunization with RNA adjuvant (G3), and G7, which was intranasally immunized with RNA adjuvant and vaccine, showed higher IgA than that of protein-immunized G6 (Figure 5F). In addition, the titer of influenza virus was measured in lung tissues obtained one week after virus challenge. Groups immunized with RNA and influenza vaccine (G3, G5, and G7) showed lower virus titers than G1. Particularly, after intranasal immunization between groups 6 and 7, virus titer in the lung was less after immunization with the RNA adjuvant than that after immunization with only the influenza vaccine without the RNA adjuvant (Figure 5G). After influenza virus challenge, G3 showed 100% survival, whereas G7 showed 90% survival (Supplementary Figure S2). IFN-γ-releasing cells were measured in splenocytes obtained one week after virus challenge, and IFN-γ-releasing T-cells were elevated in all groups containing RNA adjuvant, regardless of the immune route (Figure 6).

## 4. Discussion and Conclusions

Because MERS-CoV does not have a commercially licensed vaccine, studies to develop the vaccine are still active [24]. In the case of influenza, the vaccine is present, but the inactivated vaccine is less effective than the attenuated vaccine [25]. Generally, protein-based and/or inactivated vaccines are less immunogenic than attenuated vaccine [25,26]. Therefore, multiple rounds of inoculation to elicit protective immune responses against a pathogen is a disadvantage. Moreover, because of the relatively short duration of immunity, many studies are underway to improve this feature [27,28,29]. A potential solution is the addition of immunological adjuvant to protein-based and/or inactivated vaccines [25]. Alum has been widely used for a long time [11] but does not strongly induce the Th1 immune response. Additionally, side effects such as autoimmune/inflammatory syndrome induced by adjuvants also occur, requiring further confirmation of safety [30,31]. To compensate for these limitations, RNA adjuvants have recently been developed [19] and applied to MERS and influenza, as they did not accumulate in the body, thus lowering the risk of side effects and effectively increasing the immune response [17,18]. We found that immunization with MERS S protein with RNA adjuvant and alum synergistically induced the humoral immune response and led to balanced Th1/Th2 responses [17]. However, the immunization routes most suitable for inducing the optimal immune response with RNA adjuvant were unclear. Therefore, in this study, we tested the immune effects of intramuscular, intradermal, and intranasal injection, which are the most popular vaccination routes, with RNA adjuvant in a mouse model.

Herein, we found that intramuscular administration showed the highest IgG1, IgG2a, and NAb titers, whereas intranasal administration did not induce these antibodies. Moreover, RNA adjuvant groups (G3 and G6) more effectively induced antibody titers than others (Figure 2A,B). These results agree with those of a previous report [17]. Interestingly, only G9, which was immunized with MERS S protein, alum, and RNA adjuvant, induced IgA in the BALF (Figure 2C), which was strongly correlated with the mucosal immune system [32]. When antigen penetrates through the nasal mucosa, the mucosal immune response is induced by the interaction with APCs in nasal-associated lymphoid tissue (NALT), a representative organized lymphoid tissue of the mucosal immune system, forming a germinal center in the NALT. Clonal expansion of antigen-induced IgA+ B cells is induced to produce antigen-specific IgA [33,34,35]. This procedure may explain the induction of IgA in the groups with intranasal administration, where antigens are exposed to mucous membranes. The induction of IgA in only G9 (intranasal injection with RNA adjuvant) was dependent on the presence of RNA adjuvant, as it may affect APCs in NALT and stimulate IgA+ B cells. However, the measured influenza-specific IgA in BALF after viral challenge demonstrated that all immunization routes with RNA adjuvant induced IgA levels, and intramuscular administration induced the highest IgA level (Figure 5F). The cause of increased IgA through all injection routes may be that the virus was challenged through the mucosa. B cells were effectively activated in G3 (intramuscular injection with vaccine, alum, and RNA adjuvant), which originally had high antibody titer (Figure 2A,B), and these B cells may switch classes to generate IgA. A previous study showed that IgA class switching occurs via both T-cell-dependent and T-cell-independent mechanisms. Interestingly, T-cell-independent IgA switching occurs through TLR activation and B-cell-activating factor [36]. RNA adjuvant is a single-stranded RNA. Thus, it can activate the innate immune response through TLR7 or TLR8 [17]. Therefore, such TLR activation may contribute to IgA class switching. However, further studies are needed to confirm this.

In addition, intranasal administration protected against influenza virus challenge as much as intramuscular and intradermal administration (Figure 5E,G), even if no induction of any antibodies occurred, including HI antibody (Figure 5C,D). Therefore, the RNA adjuvant stimulates the mucosal immune response at least after pathogen infection, regardless of the immunization routes. This characteristic of RNA adjuvant is very useful for protecting against respiratory pathogen infections.

Interestingly, CD4+ T-cell activation was more effectively increased through intranasal administration compared to other routes (Figure 3). Activation of CD4+ T-cells with intranasal immunization with RNA adjuvant may occur because of the relationship between IgA induction and the RNA adjuvant. Mucosal immunity induces antigen-specific Th1 or Th2 immune responses, depending on the nature of the antigen, adjuvant, and antigen delivery vehicle used [37,38,39]. Figure 4 shows that RNA adjuvant can recruit mDCs, pDCs, and macrophages. Thus, as the RNA adjuvant was exposed to antigen through mucous membranes, it induced CD4+ T-cell activation (Figure 3), by recruiting APCs, particularly DCs in NALT [16]. In addition, RNA adjuvant may induce mucosal immunity-specific factors, such as M cells present in NALT through mucosal immunity [40]. Further studies of the correlation between ssRNA and mucosal immunity may provide insight into the cause of these effects.

According to Figure 6, IFN-γ-producing T-cells, which may play a role in protecting against virus infection [41], were increased in the RNA adjuvant formulated influenza vaccine. Thus, the RNA adjuvant increases IFN-γ-producing T-cells, contributing to the antiviral effect. 

Taken together, ssRNA used in this study can be combined with protein-based and inactivated vaccines as an adjuvant to boost the immune response, including NAbs, antigen-specific IgA, and CD4+ T-cell activation. Although we used two different vaccine types, intramuscular immunization was found to be the most effective method for inducing the humoral immune response and enhancing the Th1 response to some extent, whereas intranasal immunization was most effective for inducing mucosal immune response and CD4+ T-cell activation, at least with RNA adjuvant. Further studies are required to investigate the immune effects of RNA adjuvant according to the immunization route. 

## Supplementary Materials

The following are available online at https://www.mdpi.com/1999-4923/12/5/441/s1. Figure S1: (**A**) Group design for Middle East respiratory syndrome (MERS) spike (S) protein. (**B**) Immunization schedule. (**C**) Titers of neutralizing serum antibody against MERS-CoV in immunized mice by plaque reduction neutralizing assay. Figure S2: (**A**) Overall study design. (**B**) Survival of mice after challenge with influenza virus.
