# Supplementary material for: MERS-CoV Spike Protein Vaccine and Inactivated Influenza Vaccine Formulated with Single Strand RNA Adjuvant Induce T-Cell Activation through Intranasal Immunization in Mice
Source: Pharmaceutics. 2020 May 10;12(5):441. doi: 10.3390/pharmaceutics12050441 (PMC7284860; doi:10.3390/pharmaceutics12050441)
Supplement: Supplementary file 1 [file pharmaceutics-12-00441-s001.pdf]

pharmaceutics

Supplementary Materials: MERS-CoV Spike Protein Vaccine and Inactivated Influenza Vaccine Formulated with Single Strand RNA Adjuvant Induce T-cell Activation through Intranasal Immunization in Mice

Hye-Jung Kim, Hye Won Kwak, Kyung Won Kang, Yoo-Jin Bang, Yu-Sun Lee, Hyeong-Jun Park, Jae-Yong Kim, Hyo-Jung Park, Kyung-Ah Hwang, Sang-Myeong Lee and Jae-Hwan Nam

1. Supplementary Method

1.1. Immunization and Plaque-Reduction Neutralization Test (PRNT) for MERS-CoV

The mice were immunized twice a week at two-week intervals with the following formulations: 1 µg MERS S protein vaccine with/without 20 µg RNA adjuvant and 24 µg alum (Brentanne, Frederikssund, Denmark). Mice were injected intramuscularly, or intradermally in the upper thigh position.

The serum samples from vaccinated mice were inactivated at 56 °C for 30 min. The samples were serially diluted from 1/40 to 1/640 with serum-free medium. The virus-serum mixture was prepared by mixing 125 PFU MERS-CoV with the diluted serum samples and incubated at 37 °C for 1 h. The virus–antibody mixture was inoculated into Vero cells. The plates were incubated for 1 h at 37°C in 5% CO_2. After virus adsorption, agar overlay medium was added, and the plates were incubated at 37°C in 5% CO_2 for four days. The cells were stained with 0.4% crystal violet solution (Sigma). Plaques were counted with the naked eye. The percentage neutralization represented the reduction value, which was calculated as 100 × the number of plaques in the 100 PFU virus infected well/the number of plaques in the virus-serum mixture infected well.

1.2. Immunization for Influenza Vaccine and Challenge with Influenza Virus

The mice were immunized twice a week at two-week intervals with 0.6 µg inactivated influenza vaccine with/without 20 µg RNA adjuvant. Mice were injected intranasally or intramuscularly in the upper thigh position.

Each mouse was infected with 1.0 × 103 PFU (plaque forming units) of influenza virus (A/H1N1/California/04/09) intranasally. Influenza viruses were provided by Professor Baik-Lin Seong (Yonsei University, Seoul, South Korea).

2. Supplementary Figures

(A)

Group	G1	G2	G3	G4	G5	G6	G7	G8	G9	G10
MERS S protein	-	+	+	+	+	-	+	+	+	+
Alum	-	-	+	-	+	-	-	+	-	+
RNA adjuvant	-	-	-	+	+	-	-	-	+	+
route	I.M					I.D				

(B)

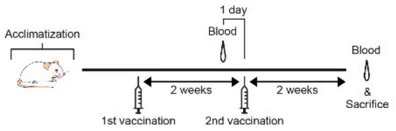

(C)

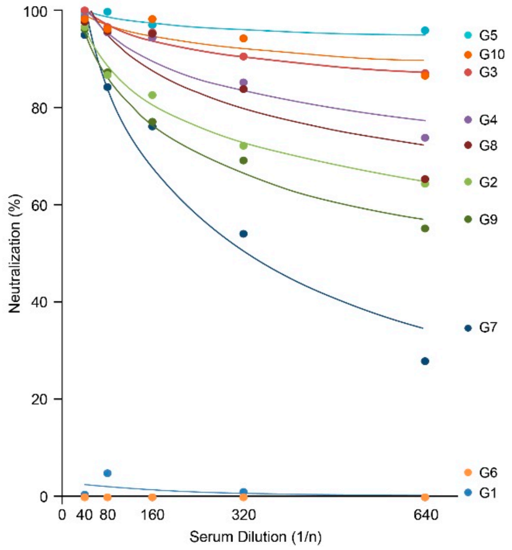

Figure S1. (**A**) Group design for Middle East respiratory syndrome (MERS) spike (S) protein. (**B**) Immunization schedule. (**C**) Titers of neutralizing serum antibody against MERS-CoV in immunized mice by plague reduction neutralizing assay.

(A)

Group	G1	G3	G7
Flu vaccine	-	+	+
RNA adjuvant	-	+	+
route		I.M	I.N

(B)

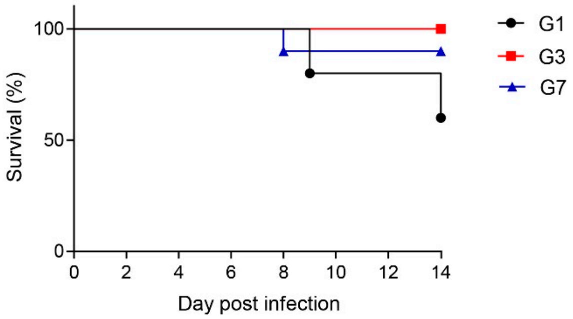

Figure S2. (**A**) Overall study design. (**B**) Survival of mice after challenge with influenza virus.
